# A Matrigel-based 3D construct of SH-SY5Y cells models the α-synuclein pathologies of Parkinson's disease

**DOI:** 10.1242/dmm.049125

**Published:** 2022-03-08

**Authors:** Zhao-Feng Li, Lei Cui, Mi-Mi Jin, Dong-Yan Hu, Xiao-Gang Hou, Shu-Shu Liu, Xiong Zhang, Jian-Hong Zhu

**Affiliations:** 1 Institute of Nutrition and Diseases, Department of Preventive Medicine, Wenzhou Medical University, Wenzhou, Zhejiang 325035, China; 2 Department of Neurology and Geriatrics, The Second Affiliated Hospital and Yuying Children's Hospital, Wenzhou Medical University, Wenzhou, Zhejiang 325027, China

**Keywords:** Parkinson's disease, 3D modeling, α-Synuclein, Lewy body, Dopaminergic neurons

## Abstract

Parkinson's disease (PD) is associated with α-synuclein-based Lewy body pathology, which has been difficult to observe in conventional two-dimensional (2D) cell culture and even in animal models. We herein aimed to develop a three-dimensional (3D) cellular model of PD to recapitulate the α-synuclein pathologies. All-trans-retinoic acid-differentiated human SH-SY5Y cells and Matrigel were optimized for 3D construction. The 3D cultured cells displayed higher tyrosine hydroxylase expression than 2D cells and improved dopaminergic-like phenotypes, as suggested by RNA-sequencing analyses. Multiple forms of α-synuclein, including monomer, and low- and high-molecular mass oligomers, were differentially present in the 2D and 3D cells, but mostly remained unchanged upon N-methyl-4-phenyl pyridine or rotenone treatment. Phosphorylated α-synuclein was accumulated, and detergent-insoluble α-synuclein fraction was observed, in the neurotoxin-treated 3D cells. Importantly, Lewy body-like inclusions were captured in the 3D system, including proteinase K-resistant α-synuclein aggregates, ubiquitin aggregation, and β-amyloid and β-sheet protein deposition. The study provides a unique and convenient 3D model of PD that recapitulates critical α-synuclein pathologies and should be useful in multiple PD-associated applications.

## Abstract (precis)

**Summary:** This study provides a convenient 3D model of Parkinson's disease (PD), which recapitulates α-synuclein pathologies in human cells and could be used to investigate PD mechanisms and screen drugs.

## INTRODUCTION

Parkinson's disease (PD) is the second most common neurodegenerative disorder after Alzheimer's disease (AD). The prevalence of PD increases dramatically with aging, and is ∼1.1% in people over 60 years of age (Cui et al., 2020). Clinical manifestations of PD include resting tremor, bradykinesia, rigidity and postural instability. Its principal pathophysiology is determined by the degeneration of dopaminergic neurons in the substantia nigra pars compacta, leading to impaired neurotransmission in the dorsolateral striatum. A number of studies have shown that PD develops from a complicated interplay between genetics and environment (Kalia and Lang, 2015).

Lewy bodies are recognized as the pathological hallmark of PD and used in Braak staging to define the disease temporal and spatial progression (Kalia and Lang, 2015). A Lewy body mainly comprises α-synuclein aggregates and other proteins such as ubiquitin, β-amyloid protein and neurofilament proteins, but its form is not monolithic. For example, it appears in a particulate form in dopaminergic neuronal bodies of the substantia nigra, an acidophilic and argyrophilic core, and a pale-staining halo in the brainstem, and is spindle- or thread-like in axons and dendrites of affected neurons (Goedert et al., 2013; Braak et al., 1994; Kuusisto et al., 2003). α-Synuclein, encoded by the gene *SNCA*, is an abundant 140-residue neuronal protein. This protein is in a dynamic equilibrium in forms of monomer, oligomer and fibril (Dehay et al., 2015). Oligomers define α-synuclein in a wide range of molecular masses, and are usually classified as low-molecular mass (LMW) and high-molecular mass (HMW) (Cullen et al., 2009; Gu et al., 2010; Lehri-Boufala et al., 2015), or as single-size molecular mass, such as aS100 (α-synuclein at 100 kDa) (Dettmer et al., 2013). Mutations in the *SNCA* gene and abnormal post-translational modification of α-synuclein can break the assembly equilibrium (Paleologou and El-Agnaf, 2012), such that A53T mutation leads to the formation of the annular and tubular structures (Lashuel et al., 2002; Sardi et al., 2013), and phosphorylation at Ser-129 promotes insoluble fibril formation *in vitro* (Fujiwara et al., 2002). Misfolded α-synuclein and its toxic aggregates, but not loss of its function, are involved in the pathogenesis and progression of PD (Makin, 2016). In recent years, a controversial prion theory of α-synuclein has gained mounting support, with increasing evidence that α-synuclein propagates throughout the brain as well as between interneurons and neurons–glial cells (Desplats et al., 2009; Hansen et al., 2011; Loria et al., 2017; Makin, 2016).

The capture of the multifactorial nature of PD is often difficult in cellular or animal models. The 1-methyl-4-phenyl-1,2,3,6-tetrahydropyridine (MPTP)-lesioned non-human primate remains the gold-standard animal model of PD and is frequently used to evaluate the effectiveness of molecules on parkinsonism and psychosis (Veyres et al., 2018). Higher MPTP dose can lead to acute dopaminergic cell loss within 1 month, while low dose usually takes 8-12 months to achieve stable parkinsonism (Bezard et al., 2001; Seo et al., 2019; Barth et al., 2020). Intraneuronal inclusions reminiscent of Lewy bodies have been described in the primate MPTP model (Dauer and Przedborski, 2003; Tieu, 2011; Forno et al., 1986). However, other animal models induced by neurotoxins or by genetic manipulations cannot consistently recapitulate this important neuropathological feature (Chia et al., 2020; Giraldez-Perez et al., 2014; Cicchetti et al., 2009), nor can the conventional two-dimensional (2D) cell culture system (Zhuang et al., 2018).

Three-dimensional (3D) models of human-derived cells have recently been extensively developed to study disease mechanisms and to screen drugs (Duval et al., 2017). Compared to the 2D cultures, these models contain mechanical structural cues and the extracellular microenvironment that brings them closer to physiological conditions (Baker and Chen, 2012). Matrigel is the ideal skeleton of a 3D model, and contains rich extracellular matrix molecules such as laminin, collagen IV and entactin. A Matrigel-based 3D model of human neural cells overexpressing familial AD mutants of β-amyloid precursor protein and/or presenilin 1 was recently reported to successfully recapitulate all key known neuronal hallmarks of AD (Choi et al., 2014). Using SH-SY5Y cells, an immortalized cell line often used for PD study and with the advantage of fast reproduction, simple culture and low cost for use in 3D cultivation, we herein provided, for the first time, a neurotoxin-induced, Matrigel-based 3D model of PD that recapitulated unique features of Lewy body-like aggregations.

## RESULTS

### Development of Matrigel- and SH-SY5Y cell-based 3D construct

All-trans-retinoic acid (RA) is often used to induce dopaminergic differentiation of SH-SY5Y cells as indicated by the expression of tyrosine hydroxylase (TH) (Xicoy et al., 2017). Different medium fetal bovine serum (FBS) concentrations (1, 2, 3, 5 and 10%) had no impact on RA (10 μM)-induced TH expression in regular 2D cultures (Fig. S1A; all statistical results listed in Table S1). Then, 1% FBS was selected for subsequent cultures to avoid over-confluency and cell aggregations. Cells used for the 3D constructs were at 6-7×10^6^ per ml based on a previous study by Choi et al. (2014) and cultured in different concentrations of Matrigel (3.5, 4.0, 4.5 and 6.0 mg/ml). Results showed that 4.5 mg/ml Matrigel conferred on cells the most appropriate spacing and the least cell aggregations (Fig. S1B), and this concentration was selected for subsequent experiments. It was previously reported that SH-SY5Y cells treated with RA followed by the addition of 12-*O*-tetradecanoylphorbol-13-acetate (TPA) [RA/(RA+TPA)] would improve dopaminergic differentiation compared to RA alone in the conventional 2D cultures (Presgraves et al., 2004). However, in the Matrigel-based 3D constructs, treatment of RA/(RA+TPA) at 10 μM/(10 μM+80 nM) resulted in dense aggregation of SH-SY5Y cells (Fig. S1C), suggesting that this type of induction is not appropriate for the 3D cultures.

**Fig. 1.**
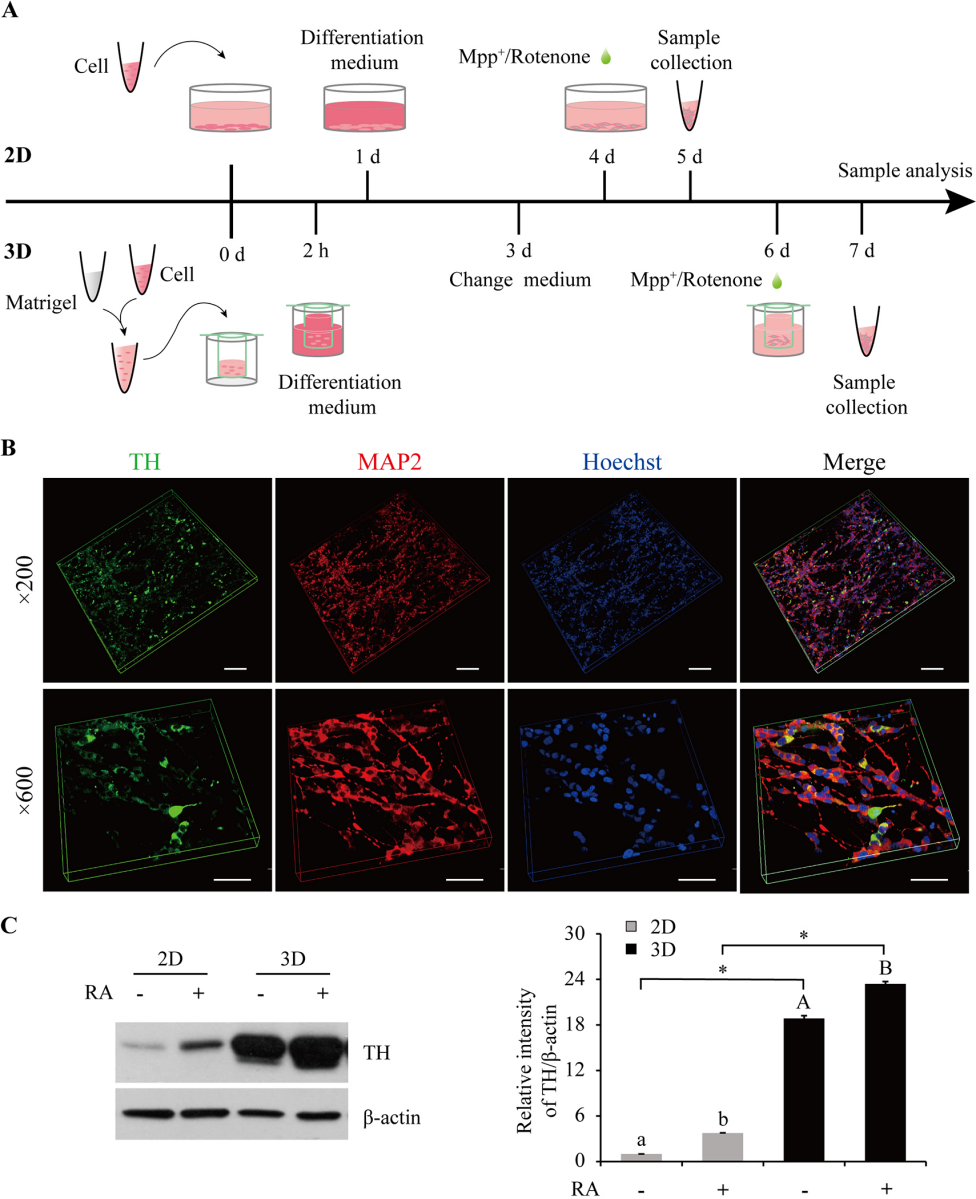
**Matrigel- and SH-SY5Y cell-based 3D constructs.** (A) Preparation and processing scheme of the 2D and 3D cultures. (B) Reconstituted 3D images of SH-SY5Y cells in a thin-layer 3D construct after 6 days in the differentiation medium. *Z*-sections were captured at 0.85-μm and 0.4-μm intervals 36 and 58 consecutive times, respectively, for the ×200 and ×600 magnifications. Green, TH; red, MAP2; blue, nuclei. Scale bars: 50 μm. (C) TH expression in the 2D and 3D cultures with or without RA induction. Protein levels were quantified and normalized to their respective β-actin levels. Values were expressed relative to the 2D cells without RA treatment, which was set as 1. Data are means±s.e.m., *n*=3. Statistical analyses were performed using factorial ANOVA. Different letters in the same casing and * between the indicated groups represent *P*<0.05. MAP2, microtubule-associated protein 2; RA, all-trans-retinoic acid; TH, tyrosine hydroxylase.

As a result, 6-7×10^6^ per ml SH-SY5Y cells, 4.5 mg/ml Matrigel, and a differentiation medium containing 1% FBS and 10 μM RA were used to establish the 3D culture, with a culture time of 6 days. The 2D counterpart showed high expression of TH during 2-4 days in the differentiation medium (Fig. S1D), and thus the culture time of 3 days was selected for comparison. The 2D and 3D preparation and modeling procedures are illustrated in Fig. 1A and detailed in the Materials and Methods section. An axonal network of SH-SY5Y cells in the 3D construct was reconstituted using confocal images immunostained with TH and the neuronal marker microtubule-associated protein 2 (MAP2) (Fig. 1B). TH expression was compared between the 2D and 3D cultures with or without RA differentiation. Results showed a massive increase in TH expression in the 3D culture without RA treatment compared to the 2D culture without RA treatment (by 17.8-fold; Fig. 1C). RA treatment induced a similar increase in TH expression in the 2D and 3D cultures (Fig. 1C).

### Transcriptomic analysis of gene expression in the RA-treated 2D and 3D cultures

**Fig. 2.**
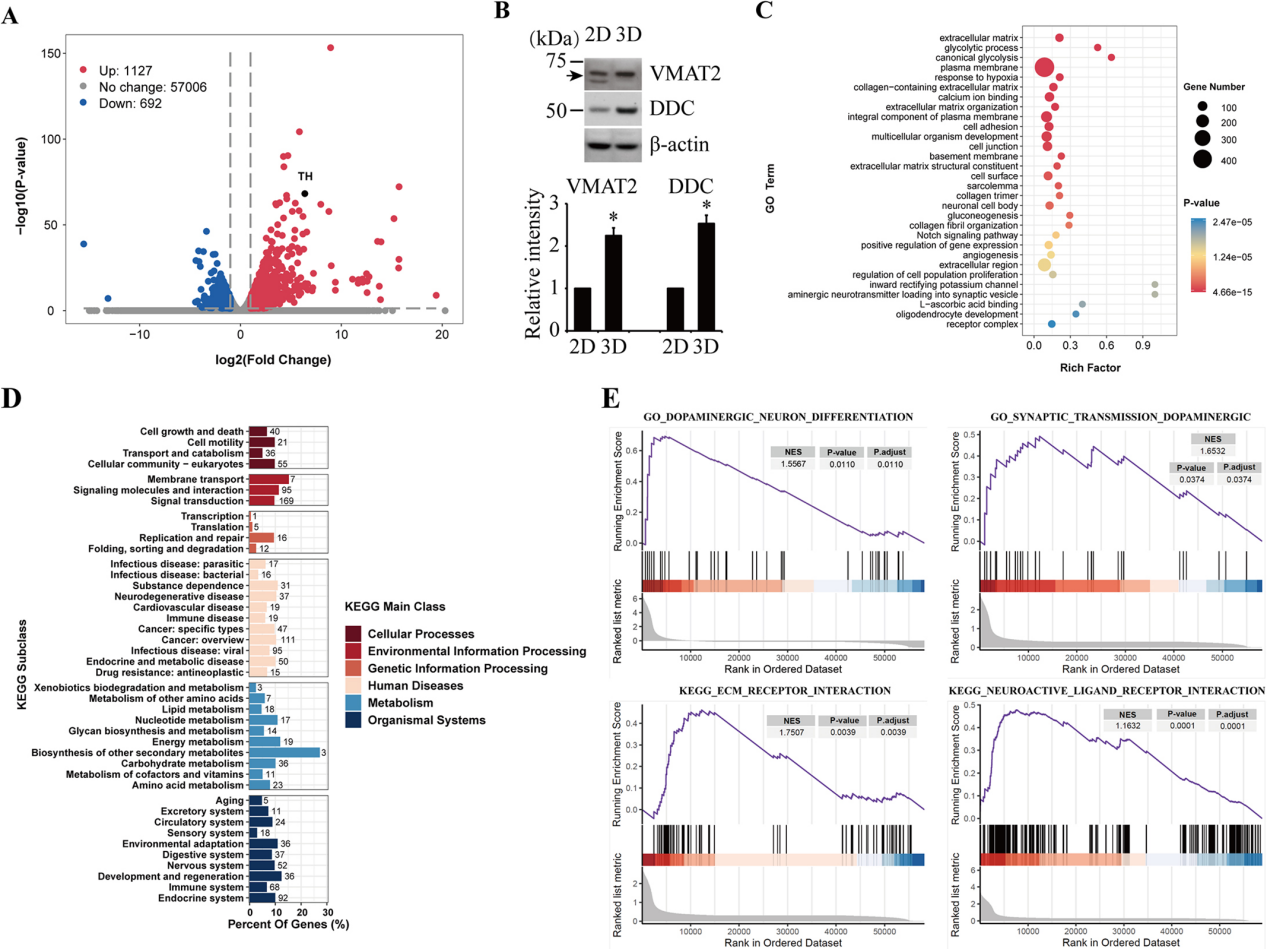
**RNA-sequencing analyses of SH-SY5Y cells in the RA-treated 2D and 3D cultures.** The data represent three replicates. (A) Volcano plot showing DEGs in the 3D cultures compared to the 2D cultures. The threshold was set at fold change >2 and *P*<0.05. (B) Western blot validation of the differential expression of *VMAT2* and *DDC* in the RA-treated 2D and 3D cultures. Protein levels were quantified and normalized to their respective β-actin levels. Values were expressed relative to the 2D cells, which was set as 1. Data are means±s.e.m., *n*=3. Statistical analyses were performed using unpaired two-tailed Student's *t*-test. *P<0.05. (C) GO analysis of the DEGs. (D) KEGG analysis of the DEGs. (E) GSEA of the RNA-sequencing data in dopaminergic neuron differentiation, dopaminergic synaptic transmission, ECM receptor interaction and neuroactive ligand–receptor interaction. DDC, dopa decarboxylase; DEGs, differentially expressed genes; ECM, extracellular matrix; GO, gene ontology; GSEA, gene set enrichment analysis; KEGG, Kyoto Encyclopedia of Genes and Genomes; NES, normalized enrichment score; RA, all-trans-retinoic acid; VMAT2, vesicular monoamine transporter 2.

RNA sequencing was used to analyze transcriptomic expression of differentially expressed genes (DEGs) in the 2D and 3D cultures differentiated with RA as above. Compared to the 2D cultured cells, a total of 1127 upregulated and 692 downregulated genes were identified in the 3D cultured cells (Fig. 2A; full list in Table S2). Consistent with the above changes in protein expression, the mRNA expression of *TH* was massively elevated in the 3D culture (by 81.8-fold; Fig. 2A). We could also validate the elevated expression of two additional DEGs, vesicular monoamine transporter 2 (*VMAT2*; also known as *SLC18A2*) and dopa decarboxylase (*DDC*), by western blotting for the proteins encoded by the genes (Fig. 2B). The DEGs were enriched in categories such as extracellular matrix, glycolytic process, canonical glycolysis, plasma membrane and response to hypoxia, as suggested by gene ontology (GO) biological function analysis (Fig. 2C). Kyoto Encyclopedia of Genes and Genomes (KEGG) pathway analysis suggested that the DEGs were enriched in categories such as cellular community, membrane transport, replication and repair, neurodegenerative disease, energy metabolism and nervous system (Fig. 2D). Gene set enrichment analysis (GSEA) revealed the enrichment of DEGs in dopaminergic neuron differentiation, dopaminergic synaptic transmission, extracellular matrix receptor interaction and neuroactive ligand–receptor interaction (Fig. 2E). Results of the dopaminergic synaptic signal pathway diagram of DEGs showed altered expressions in *TH* (elevated), *DDC* (elevated), *VMAT1* (also known as *SLC18A1*) and *VMAT2* (elevated), dopamine receptor D5 (*DRD5*; reduced), and electric potential and signal transduction-associated genes including calcium voltage-gated channel subunit alpha1 (*CACNA1D*; reduced) and protein kinase C gamma (*PRKCG*; elevated). Products of the first four DEGs are involved in presynaptic dopamine production and transport, while the others function in postsynaptic neurons (Fig. S2).

### MPP^+^- and rotenone-induced α-synuclein pathologies in the 2D and 3D cultures

**Fig. 3.**
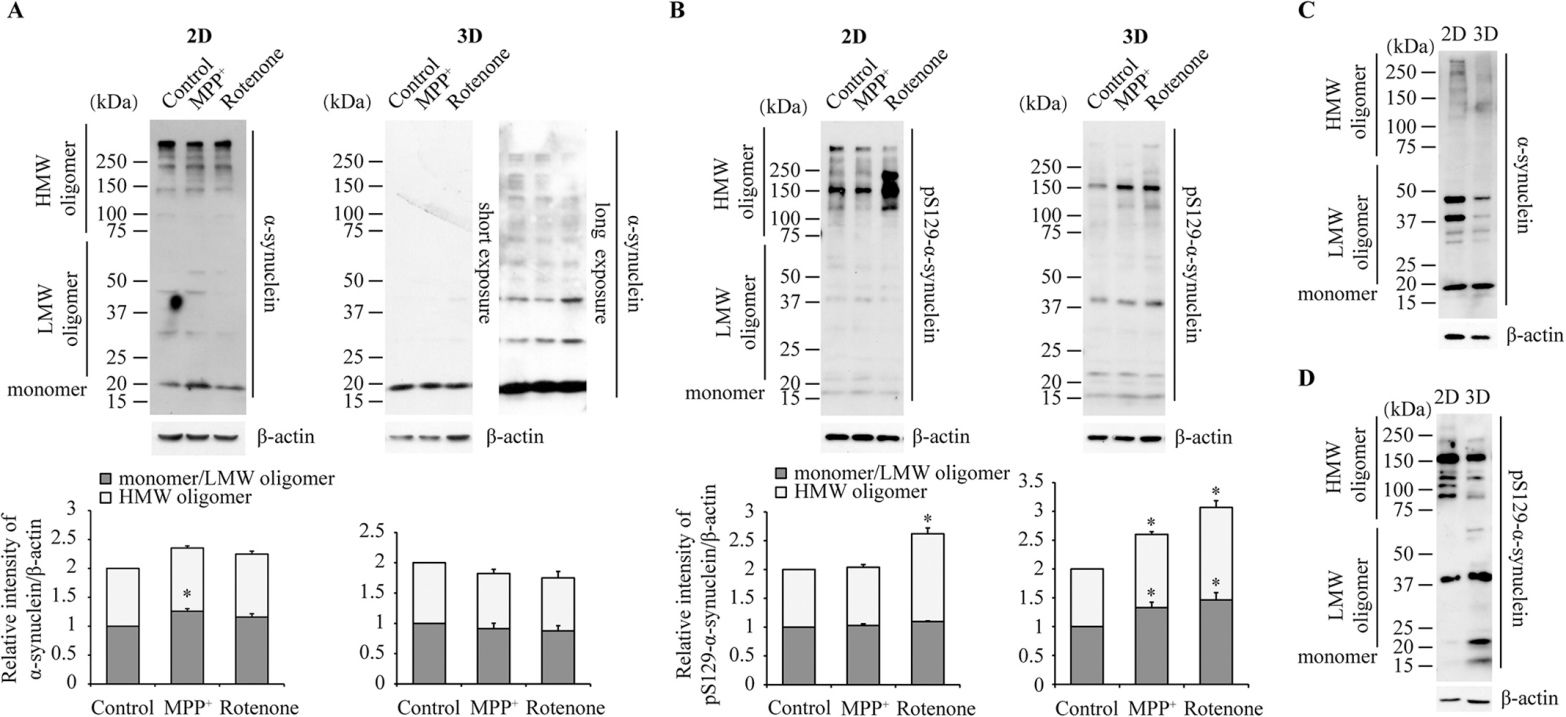
**Western blot analyses of α-synuclein and pS129-α-synuclein expression.** (A,B) Analysis of α-synuclein (A) and pS129-α-synuclein (B) in the RA-induced 2D and 3D cultures treated with 10 μM MPP^+^ or 0.5 μM rotenone for 24 h. Protein levels were quantified and normalized to their respective β-actin levels. Values were expressed relative to the control, which was set as 1. Data are means±s.e.m., *n*=3. Statistical analyses were performed using unpaired two-tailed Student's *t*-test. *P<0.05. (C,D) Baseline comparison of α-synuclein (C) and pS129-α-synuclein (D) in the RA-treated 2D and 3D cultures. As a note, we observed that α-synuclein aggregation state may vary a bit among different cell batches. Thus, 2D and 3D direct comparison should be more appropriate using cells of the same batch. HMW, high-molecular mass; LMW, low-molecular mass.

Cells of the 2D and 3D cultures were treated with classical neurotoxins, N-methyl-4-phenyl pyridine (MPP^+^) at 10 μM and rotenone at 0.5 μM, for 24 h. Western blot results for the 2D cells showed that the MPP^+^ treatment induced significant increase in levels of α-synuclein monomer and LMW oligomer (<75 kDa), but not in HMW oligomer (>75 kDa). The rotenone treatment induced no change in the α-synuclein forms. In contrast, neither MPP^+^ nor rotenone treatment changed the α-synuclein forms in the 3D culture (Fig. 3A). Western blot results for pS129-α-synuclein appeared to be different. Whereas the MPP^+^ treatment caused no changes, the rotenone treatment increased pS129-α-synuclein HMW oligomer, but not monomer/LMW oligomer, in the 2D cultures. In contrast, MPP^+^ and rotenone increased both pS129-α-synuclein forms in the 3D cultures (Fig. 3B). Comparing the baseline level in the 3D cells to that in the 2D cells, we observed that the proportion of monomer to total α-synuclein was elevated in the 3D cells (Fig. 3C). pS129-α-synuclein was largely present as a HMW oligomer in the 2D cells, while the forms existed roughly in similar abundance in the 3D cells (Fig. 3D).

**Fig. 4.**
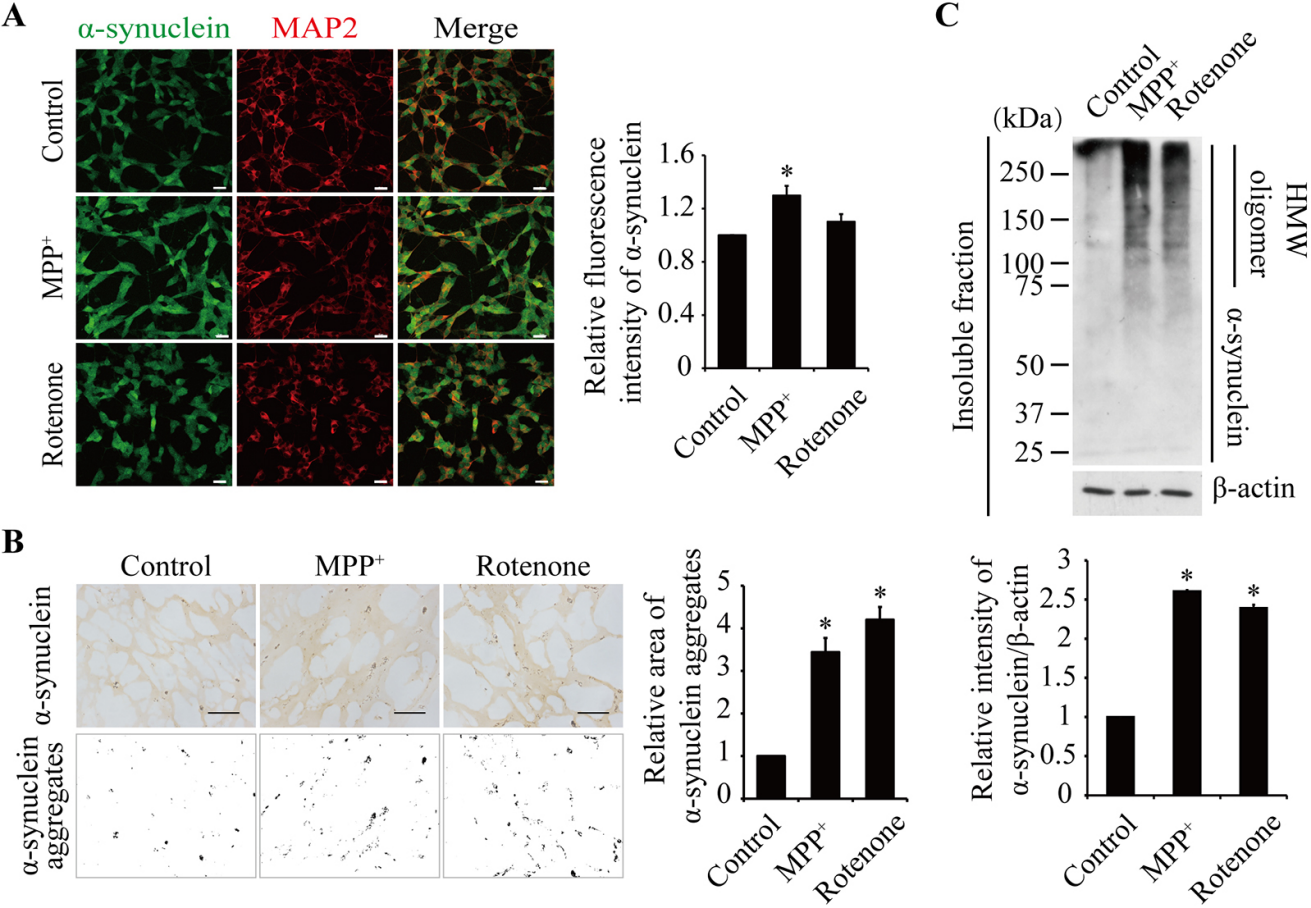
**Analyses of α-synuclein in the 2D and 3D cultures treated with 10 μM MPP^+^ or 0.5 μM rotenone for 24 h.** (A) Immunofluorescence staining of the 2D cultures. Green, α-synuclein; red, MAP2. Scale bars: 25 μm. (B) Immunohistochemical (IHC) staining of sections of the 3D cultures. The images were contrasted for quantitative analysis of α-synuclein aggregates using ImageJ software. Scale bars: 100 μm. (C) Western blot analyses of insoluble α-synuclein fraction in the 3D cultures. Protein levels were quantified and normalized to β-actin. Values were expressed relative to the control, which was set as 1. Data are means±s.e.m., *n*=3. Statistical analyses were performed using unpaired two-tailed Student's *t*-test. *P<0.05.

Immunofluorescence analysis of α-synuclein in the 2D cultures showed that the protein was significantly elevated by the MPP^+^ treatment, but not by rotenone (Fig. 4A), which is consistent with the above western blot analysis results. However, immunofluorescence analysis of α-synuclein in the 3D constructs showed high background staining, probably resulting from the Matrigel nonspecific binding (Fig. S3). We thus used immunohistochemical (IHC) staining for the 3D constructs, from which there were much less background signal. Results showed an elevated aggregation area of α-synuclein immunopositivity upon treatment with either MPP^+^ or rotenone (Fig. 4B). We also prepared detergent-insoluble fractions and found that the insoluble α-synuclein level was increased by both treatments, mainly in the form of HMW oligomer (Fig. 4C). However, β-amyloid protein was not detectable in the fractions. Similar to α-synuclein (Fig. 3A), β-amyloid protein levels were not altered by the neurotoxin treatments in 3D cultures (Fig. S4A).

### Intraneuronal aggregation of Lewy body α-synuclein in the 3D cultures administered MPP^+^ and rotenone

**Fig. 5.**
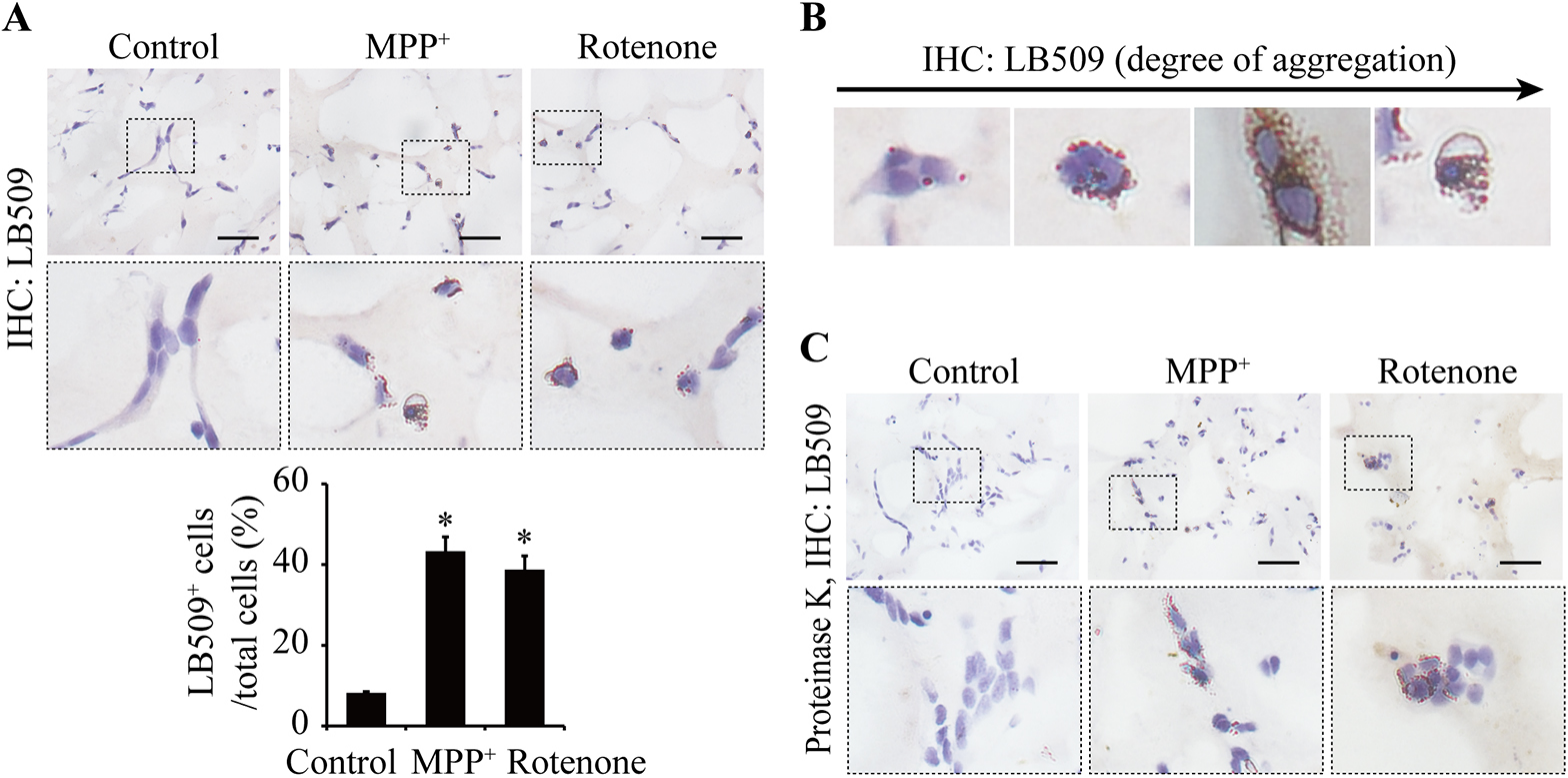
**Analyses of α-synuclein aggregation in the 3D cultures treated with 10 μM MPP^+^ or 0.5 μM rotenone for 24 h.** (A) IHC staining of LB509-α-synuclein. (B) Representative images of LB509-α-synuclein-positive cells with different degrees of aggregation. (C) IHC staining of LB509-α-synuclein after digestion with 50 μg/ml proteinase K. Scale bars: 50 μm. LB509-α-synuclein-positive cells and total cells were counted from three to four random areas. *n*=3. Data are means±s.e.m., *n*=3. Statistical analyses were performed using unpaired two-tailed Student's *t*-test. *P<0.05.

**Fig. 6.**
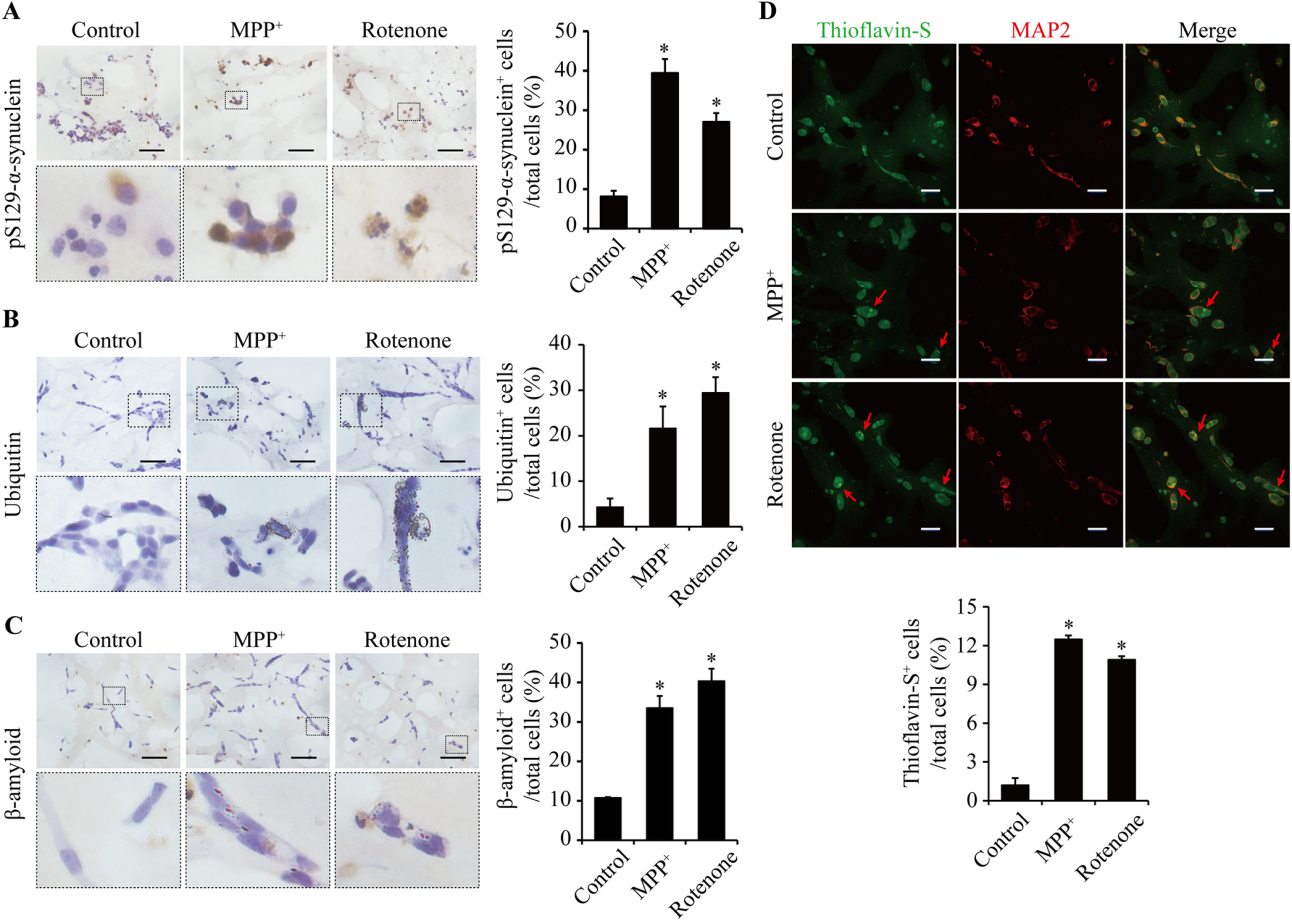
**Aggregation and deposition analyses in the 3D cultures treated with 10 μM MPP^+^ or 0.5 μM rotenone for 24 h.** (A-C) IHC staining of pS129-α-synuclein (A), ubiquitin (B) and β-amyloid (C). Scale bars: 50 μm. (D) Thioflavin-S staining for β-sheet protein. Scale bars: 25 μm. The positive cells and total cells were counted from three random areas. *n*=3. Data are means±s.e.m., *n*=3. Statistical analyses were performed using unpaired two-tailed Student's *t*-test. *P<0.05.

Lewy pathology was detected by a standard histological methodology, including the application of LB509-α-synuclein, an antibody raised against Lewy body α-synuclein, and proteinase K digestion (Ip et al., 2017; Goedert et al., 2013). IHC staining of LB509-α-synuclein showed a large and significant increase in intracytoplasmic aggregation dots in the SH-SY5Y cells of the 3D constructs following treatment with 10 μM MPP^+^ or 0.5 μM rotenone for 24 h (Fig. 5A). The intracytoplasmic aggregations appeared to be of different degrees of density, from light, medium, high to condense around the nuclei (Fig. 5B). To demonstrate that these aggregates are degradation resistant, the sections were pretreated with proteinase K. IHC results showed that the MPP^+^- and rotenone-induced aggregation dots remained in significant amount after the treatment (Fig. 5C). However, western blot analysis did not detect any bands of proteinase K-resistant α-synuclein (Fig. S4B). The 3D constructs were then analyzed for phosphorylated α-synuclein, ubiquitin and β-amyloid protein by IHC staining, and β-sheet protein by thioflavin-S staining. Like results for the LB509-α-synuclein staining, treatment of the 3D constructs with MPP^+^ or rotenone led to significant increase in the number of cells accumulated with phosphorylated α-synuclein expression (Fig. 6A), ubiquitin aggregation (Fig. 6B), β-amyloid protein expression (Fig. 6C) and β-sheet protein deposition (Fig. 6D), which together suggest a Lewy body-like formation.

## DISCUSSION

An appropriate human 3D cellular model of disease may provide a unique and convenient platform for studying molecular mechanisms and screening therapeutic compounds. As noted earlier, neurofibrillary tangles, a hallmark pathology of AD, have been recapitulated in a 3D cellular model overexpressing familial AD mutants (Choi et al., 2014). Herein, we provide a convenient 3D model of PD based on Matrigel, differentiated SH-SY5Y cells and neurotoxins, which remarkably recapitulates α-synuclein pathologies, including elevated α-synuclein phosphorylation and Lewy body-like inclusions.

Two-dimensional cell culture is arguably too simple and overlooks many parameters that are important for reflecting cell and tissue physiology or pathology. These include interactions between cells and matrix, communication between adjacent cells and mechanical connections. On the other hand, *in vivo* animal models are time consuming, such as for pharmaceutical compound screening, and may have low predictability for human diseases (Van Der Worp et al., 2010; Leist and Hartung, 2013; Warren et al., 2015). For instance, a binding mapping of four conserved transcription factors in human and mouse hepatocytes suggests that 41-89% of their binding events are species specific (Odom et al., 2007). Moreover, genomic responses in mouse models correlate poorly with the human conditions of inflammatory diseases such as trauma, burns and sepsis (Seok et al., 2013). The poor predictability may also result from bias in animal studies, such as randomization, blinded outcome assessment and sample size calculation (Van Der Worp et al., 2010). A 3D culture system using human cells of wild type or mutants may thus act as a bridge in between, where cells are subjected to mechanical, structural and surrounding cues and may better reproduce physiological conditions and/or pathological changes of diseases (Baker and Chen, 2012).

Indeed, the 3D culture brings SH-SY5Y cells a few good indicators to be relatively closer to a dopaminergic neuron-like state. The 3D culture itself was associated with a high-fold increase in TH expression, which is usually not achievable by the existing cell differentiation protocols, including RA, RA/TPA or RA/brain-derived neurotrophic factor (BDNF) (Kovalevich and Langford, 2013; Presgraves et al., 2004; Xicoy et al., 2017). RNA-sequencing-based DEGs further suggest that cells in the 3D culture better resemble the *in vivo* dopaminergic phenotype as manifested by results of the GO, KEGG and GSEA analyses. Indeed, extracellular matrix proteins are enriched in the scaffold Matrigel, which together with mechanostructural cues may be crucial for induction of proper synaptic structures, functional axonal vesicle transport and sustainable neuronal morphology (Agholme et al., 2010; Baker and Chen, 2012; Hughes et al., 2010). The DEGs also provide molecular insights for further exploring how this transition occurs. In addition, human embryonic stem cells and inducible pluripotent stem cells have also been used for 3D model construction (Choi et al., 2014; Duval et al., 2017; Jo et al., 2016). Stem cells have the advantage of differentiating to desired cell types and being closely like primary cells but are relatively more expensive and time consuming to culture. In contrast, immortalized cells are cost effective, usually fast growing and easier to manipulate. Both cell types are worth 3D modeling with if *in vivo* pathological or physiological key features can be recapitulated.

When comparing, at the normal state (i.e. untreated), the proportion of total or phosphorylated α-synuclein, it shifts more to the monomer/LMW oligomer state in the 3D model than in the 2D culture. It remains unclear how the shifts occur, but suggests that the 3D model is closer to the *in vivo* physiological state based upon the premise that greater levels of aggregation are closer to the disease state (which may be in debate). After that, the neurotoxin treatments lead to a building of the disease state. The 3D model also appears superior to the 2D culture, with a better and more sophisticated manifestation of the disease state. The most exciting and meaningful finding is the recapitulation of α-synuclein pathologies of PD in the 3D model upon neurotoxin induction, including accumulation of the phosphorylated α-synuclein and detergent-insoluble α-synuclein fraction, and the Lewy body-like inclusions as manifested by the intracytoplasmic LB509-stained α-synuclein aggregates, resistance to proteinase K, ubiquitin-stained aggregates, β-amyloid and thioflavin-S-stained β-sheet protein deposits.

As a note, unlike the α-synuclein phosphorylation, the neurotoxin-induced α-synuclein resistance to proteinase K appears to be solely observable by IHC analysis, but not by western blotting. Theoretically, the availability of α-synuclein for digestion by proteinase K under conditions used for IHC analysis is limited, hence digesting lysates and analyzing by western blotting provide a more sensitive assay for protease resistance. According to literature searches and to the best of our knowledge, it seems that western blot analysis of proteinase K resistance mostly identifies exogenous α-synuclein preformed fibrils (Guo et al., 2013; Pujols et al., 2018), or requires large amount of sample such as ∼1 g of human brain extracts (Neumann et al., 2002). Instead, IHC analysis is more widely used for detection of endogenous resistant α-synuclein (Spencer et al., 2016; Ip et al., 2017; Svarcbahs et al., 2018). Indeed, even limited aggregation dots from IHC analysis are observable under a microscope, but the amount may not be enough to reach western blot detection limit. Nonetheless, IHC analysis has its own limitations, such as how specific the antibody is and how fully proteinase K can penetrate the tissue slides for aggregate digestion.

Abnormally phosphorylated α-synuclein has been observed in multiple cellular and animal models (Lou et al., 2010; Sugeno et al., 2008; Gorbatyuk et al., 2008; Kim et al., 2006), which promotes insoluble fibril formation (Fujiwara et al., 2002), and is associated with pathological α-synuclein deposits contained within Lewy bodies and Lewy neurites (Lee et al., 2014; Spillantini et al., 1997). In contrast, the Lewy body-like inclusions are mostly observed in 2D, 3D or *in vivo* models induced by exogenously added preformed α-synuclein fibrils (Mahul-Mellier et al., 2020; Uemura et al., 2018; Volpicelli-Daley et al., 2014; Taylor-Whiteley et al., 2019), but are hardly observed in neurotoxin-based models, aside from monkeys, as noted earlier (Tieu, 2011; Dauer and Przedborski, 2003; Forno et al., 1986; Chia et al., 2020; Giraldez-Perez et al., 2014; Cicchetti et al., 2009; Zhuang et al., 2018). A 3D organoid system derived from stem cells containing a G2019S mutation in leucine-rich repeat kinase 2 (*LRRK2*) displayed an accumulation of pS129-α-synuclein, with no change in total α-synuclein, which is consistent with our observation. However, the Lewy body pathology was not reported to be recaptured in this organoid (Kim et al., 2019). In addition, our model uses neurotoxins as a variable to induce α-synuclein dysfunction. Given potential disconnection between toxin models and idiopathic PD, using either *SNCA* overexpression (modeling the duplication and triplication mutations observed in human populations) or point mutations would be an alternative disease modeling approach.

In conclusion, the current study provides a novel neurotoxin-based 3D human cellular model of PD exhibiting accumulation of α-synuclein phosphorylation and Lewy body-like inclusions. The model is convenient to construct, manipulate and observe, and should prove useful in investigating PD mechanisms and screening drugs targeting α-synuclein pathologies.

## MATERIALS AND METHODS

### Cells and reagents

SH-SY5Y cells (American Type Culture Collection, Manassas, VA, USA) were cultured in Dulbecco's modified Eagle medium (DMEM) supplemented with 10% fetal bovine serum (FBS; 086-150, Wisent) and 1% penicillin/streptomycin (Gibco) and maintained in a humidified incubator at 37°C with 5% CO_2_. RA (PHR1187), MPP^+^ (M0896), rotenone (R8875) and TPA (P8139) were purchased from Sigma-Aldrich. Primary antibodies used in this study were anti-α-synuclein (6210789, BD Biosciences), anti-pS129-α-synuclein (phosphorylated α-synuclein at serine 129; ab59264, Abcam), anti-LB509-α-synuclein (180215, Thermo Fisher Scientific), anti-β-amyloid (803004, BioLegend), anti-β-actin (4970, Cell Signaling Technology), anti-DDC (10166-1, Proteintech), anti-MAP2 (4542, Cell Signaling Technology), anti-TH (22941, ImmunoStar), ubiquitin (ab7780, Abcam) and anti-VMAT2 (20873-1-AP, Proteintech).

### 3D model construction

SH-SY5Y cells were differentiated in a differentiation medium as optimized in the Results section. The cells were embedded in the Matrigel matrix containing 60% laminin, 30% collagen IV and 8% entactin (356237, Corning) to generate 3D constructs. In detail, Matrigel was taken out from −80°C, and placed in a 4°C refrigerator 2 h before use. SH-SY5Y cells were collected at a confluency of 70-80%, resuspended in DMEM with 1% FBS and placed on ice. Cold Matrigel was added to the cell suspension to obtain final concentrations of Matrigel and cells at 4.5 mg/ml and 6-7×10^6^/ml, respectively. The mixture was prepared on ice and vortexed for 30 s. For thin-layer 3D blocks (used for 3D reconstruction of confocal imaging), 400 μl of the mixture was dispensed into a glass-bottom dish (D35-20-1-N, Cellvis) using prechilled pipettes. For thick-layer 3D blocks (used for all other experiments), 300 μl of the mixture was dispensed into a cell culture insert (MCEP24H48, Millipore) in 24-well plates. The constructs were incubated in a CO_2_ incubator at 37°C for 2 h, then prewarmed differentiation medium was added to the dishes or inserts: 3 ml for thin-layer 3D cultures, and 0.5 ml to the insert and 1 ml to the surrounding well for thick-layer 3D cultures. The constructs were then cultured for 6 days before use. The differentiation medium was refreshed every 3 days.

### RNA sequencing

Total RNA of SH-SY5Y cells in the RA-treated 2D and 3D cultures was extracted using RNAiso Plus (9108, Takara) according to the manufacturer's protocol. RNA sequencing was performed on an Illumina X10 at LC Sciences (Hangzhou, China). Prior to assembly, the low-quality reads that contained sequencing adaptors, sequencing primer and nucleotide with Q quality score lower than 20 were removed. HISAT package was used to map reads of samples to the *Homo sapiens* reference genome at http://genome.ucsc.edu/. The mapped reads of each sample were assembled using StringTie and merged to reconstruct a comprehensive transcriptome using Perl scripts. After that, StringTie was used to calculate expression levels of the mRNAs by calculating fragments per kilobase per million mapped reads (FPKM). The differentially expressed mRNAs and genes were selected by Ballgown with fold change >2 and with statistical significance *P*<0.05. Bioinformatic analyses were performed using the OmicStudio tools at https://www.omicstudio.cn/tool. The dopaminergic synaptic pathway diagram was mapped using KEGG mapping tools at https://www.genome.jp/kegg/mapper.html.

### Cell lysate preparation and western blotting

Cells in thick-layer 3D blocks were recovered following incubation with Cell Recovery Solution (354253, Corning) at 4°C for 1-2 h, and then centrifuged at 330 ***g*** for 5 min at 4°C. For total cell lysates, cells from either 2D or 3D cultures were lysed in a buffer containing 60 mM Tris-HCl, pH 6.8, 5% glycerol and 2% SDS. For insoluble fraction preparation, cells were first lysed in Tris-buffered saline (TBS; 50 mM Tris, 150 mM NaCl, pH 7.5) supplemented with 1% Triton X-100, protease inhibitor cocktail (7012L, Cell Signaling Technology) and 1 mM phenylmethane sulfonyl fluoride (PMSF; ST506-2, Beyotime). After sonication using a fine probe (0.5 s pulse at an amplitude of 20%, ten times), cell lysates were incubated on ice for 30 min and centrifuged at 100,000 ***g*** for 30 min at 4°C. The pellet was washed with the lysis buffer, sonicated as above again, and centrifuged for another 30 min at 100,000 ***g***. After removing the supernatant, the pellet was resuspended in TBS supplemented with 2% SDS, protease inhibitor cocktail and 1 mM PMSF, and sonicated as above 15 times. Cell lysates were then boiled for 10 min. Total protein concentration was measured using a BCA kit (ST023, Beyotime) after centrifugation. Western blotting was performed as previously described (Liu et al., 2014). For α-synuclein analysis, the membrane was pre-fixed with 0.4% paraformaldehyde (PFA) for 30 min before blocking with 5% milk. Membranes were probed with primary antibodies against α-synuclein (1:1000), pS129-α-synuclein (1:1000), β-amyloid (1:1000), VMAT2 (1:1000), DDC (1:1000), TH (1:1000) or β-actin (1:2000). Anti-mouse (7076) and anti-rabbit (7074) secondary antibodies, and LumiGLO Reagent and Peroxide chemiluminescence detection kit (7003) were purchased from Cell Signaling Technology.

### Immunofluorescence staining

For 2D cultures, cells were fixed in 4% PFA for 30 min, followed by permeabilization in 0.2% Triton X-100 for 15 min. Samples were then blocked with 5% bovine serum albumin (BSA; P0010, Beyotime) for 1 h at room temperature. The washing buffer was phosphate-buffered saline (PBS). For thin-layer 3D blocks, the procedure was slightly modified from that of Kim et al. (2015). In brief, cells were fixed in 4% PFA containing 0.1% glutaraldehyde for 1 h, followed by permeabilization in 0.5% Triton X-100 for 1 h at room temperature. Samples were then blocked with 5% BSA overnight at 4°C. The washing buffer was TBS containing 0.2% (v/v) Tween-20. Thereafter, samples were incubated with primary antibodies against α-synuclein (1:500), MAP2 (1:200) and/or TH (1:500) at 4°C overnight, followed by incubation with Alexa Fluor 555 anti-rabbit (A21428, Thermo Fisher Scientific) and/or Alexa Fluor 488 anti-mouse (A11001, Thermo Fisher Scientific) secondary antibodies at room temperature for 2 h. Cells were then counterstained with Hoechst 33342 (H3570, Thermo Fisher Scientific) for 5 min at room temperature and mounted with an anti-fade mounting medium (P0126, Beyotime). The fluorescence images were captured and reconstituted using a Nikon confocal microscope (C2si).

### Sectioning of thick-layer 3D blocks

The thick-layer 3D blocks were fixed in 4% PFA containing 0.1% glutaraldehyde for 2 h, dehydrated in 30% sucrose for 24 h, and then subjected to optimal cutting temperature (OCT) compound (80202-0001, CellPath) embedding and frozen sectioning at 15 µm thickness using a Leica microtome (CM1860) following the manufacturer's procedure. Next, sections were incubated with PBS to remove the OCT compound at room temperature, followed by incubation with 3% (v/v) H_2_O_2_ for 20 min to block endogenous peroxidase activities.

### IHC staining

The 3D sections were permeabilized in 0.5% Triton X-100 for 15 min and blocked with 5% BSA for 1 h at room temperature. The washing buffer was PBS. The sections were then incubated with primary antibodies against LB509-α-synuclein (1:500), pS129-α-synuclein (1:200), β-amyloid (1:500) or ubiquitin (1:500) at 4°C overnight, followed by incubation with anti-mouse or anti-rabbit Ig HRP polymer conjugates (PV-6002 and PV-6001, respectively, Zhongshan Golden Bridge) at room temperature for 1 h. Signals were developed using 3,3′-diaminobenzidine peroxidase substrate kits (ZLI-9018, Zhongshan Golden Bridge). Nuclei were counterstained with Hematoxylin (C0107, Beyotime).

### Proteinase K digestion

To detect proteinase K-resistant α-synuclein aggregates, the 3D sections were treated with 50 μg/ml proteinase K prepared in a buffer containing 10 mM Tris-HCl (pH 7.4) and 100 mM NaCl at 37°C for 30 min. The sections were then subjected to IHC processing as described above using the LB509-α-synuclein antibody.

### Thioflavin-S staining

Thioflavin-S indicates β-sheets and is often used in β-amyloid and Lewy body pathologies (Urbanc et al., 2002; Mclellan et al., 2003; Li et al., 2010). The 3D sections were incubated in PBS to remove the OCT compound, then permeabilized in 0.2% Triton X-100 for 15 min and blocked with 5% BSA for 1 h at room temperature. The sections were then incubated with 0.05% thioflavin-S for 5 min at room temperature, followed by cleaning with 50% anhydrous ethanol three times for 5 min each.

### Statistical analysis

Statistical differences were evaluated using unpaired two-tailed Student's *t*-test, factorial analysis of variance (ANOVA), or one-way ANOVA followed by Tukey's post hoc test. Data were expressed as means±s.e.m. from at least three independent experiments. Differences were considered statistically significant at *P*<0.05.

## Supplementary Material

Supplementary information
