# Supplementary material for: A Matrigel-based 3D construct of SH-SY5Y cells models the α-synuclein pathologies of Parkinson's disease
Source: Dis Model Mech. 2022 Mar 8;15(3):dmm049125. doi: 10.1242/dmm.049125 (PMC8922027; doi:10.1242/dmm.049125)
Supplement: Supplementary information [file dmm-15-049125-s1.pdf]

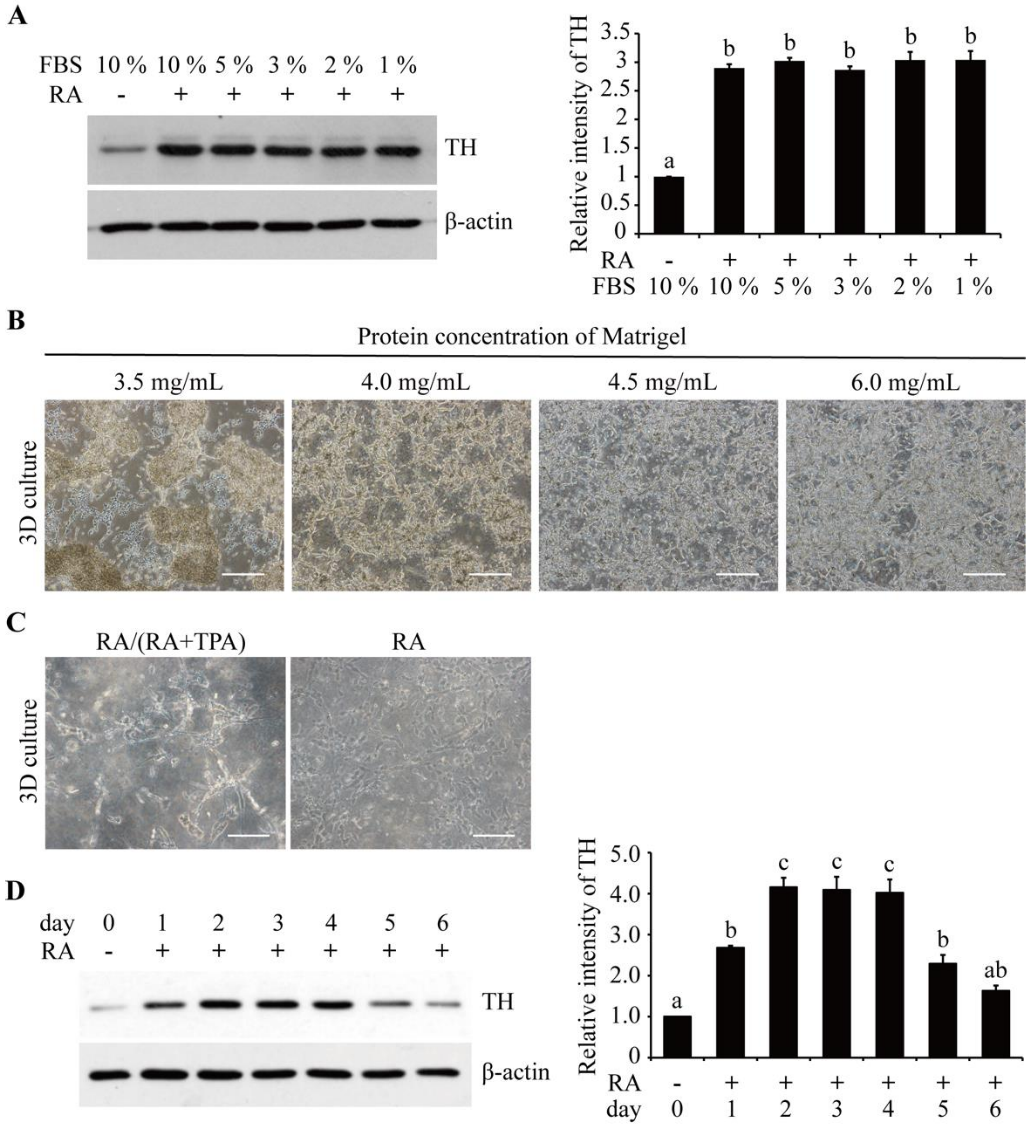

Fig. S1. Optimization of SH-SY5Y cell cultures. (A) SH-SY5Y cells were 2D cultured and treated with 10 µM RA for 3 days in media containing different concentrations of FBS. (B) Bright-field images of SH-SY5Y cells in 3D constructs with different concentrations of Matrigel. The constructs were cultured for 6 days in a differentiation medium containing 10 µM RA and 1% FBS. Bar size, 200 µm. (C) Bright-field images of cells in 3D constructs with 4.5 mg/mL of Matrigel. The constructs were cultured in a medium containing 1% FBS with two different differentiation inducers, that is, 10 µM RA for 6 days (RA) or 10 µM RA for 3 days followed by 10 µM RA and 80 nM TPA for 3 days [RA/(RA+TPA)]. Bar size, 100 µm. (D) Cells were 2D cultured and treated with 10 µM RA for different days in a medium containing 1% FBS. TH levels were quantified and normalized to their respective β-actin levels. Values were expressed relative to the one without RA treatment, which was set as 1. Data are means ± SE, n = 3. Statistical analyses were performed using one-way ANOVA, followed by Tukey's post hoc test. Different letters indicate $p < 0.05$. FBS, fetal bovine serum; RA, all-trans-retinoic acid; TH, tyrosine hydroxylase; TPA, 12-*O*-tetradecanoylphorbol-13-acetate.

A

The differentially expressed genes in dopaminergic synaptic signal pathway				
**Up-regulated genes (n=12)**				
Gene Symbol	Gene Full Name	Encoded Protein	Fold change	*P* value
TH	tyrosine hydroxylase	TH	82.77	6.20×10^{-69}
SLC18A1	solute carrier family 18 member A1	VMAT1	11.23	4.13×10^{-63}
SLC18A2	solute carrier family 18 member A2	VMAT2	5.00	2.14×10^{-7}
KCNJ5	potassium voltage-gated channel subfamily J member 5	GIRK4	3.89	4.92×10^{-4}
DDC	dopa decarboxylase	DDC	3.61	2.19×10^{-21}
PPP1R1B	protein phosphatase 1 regulatory inhibitor subunit 1B	DARPP32	3.45	4.68×10^{-2}
CALY	calcyon neuron specific vesicular protein	Calcyon	2.64	2.92×10^{-6}
GNG3	G protein subunit gamma 3	Gγ3	2.51	3.16×10^{-10}
GNAS	GNAS complex locus	$G_s\alpha$	2.44	4.05×10^{-13}
PRKCG	protein kinase C gamma	PKCγ	2.44	1.85×10^{-4}
ITPR3	inositol 1,4,5-trisphosphate receptor type 3	IP3R3	2.35	5.14×10^{-4}
GNAO1	G protein subunit alpha o1	$G_{o1}\alpha$	2.15	3.48×10^{-5}
**Down-regulated genes (n=4)**				
Gene Symbol	Gene Full Name	Encoded Protein	Fold change	*P* value
DRD5	dopamine receptor D5	D5	0.09	3.85×10^{-7}
ITPR2	inositol 1,4,5-trisphosphate receptor type 2	IP3R2	0.33	2.61×10^{-6}
CACNA1D	calcium voltage-gated channel subunit alpha1 D	$Ca_v1.3$	0.34	7.16×10^{-7}
GRIA2	glutamate ionotropic receptor AMPA type subunit 2	AMPA2	0.50	7.18×10^{-6}

B

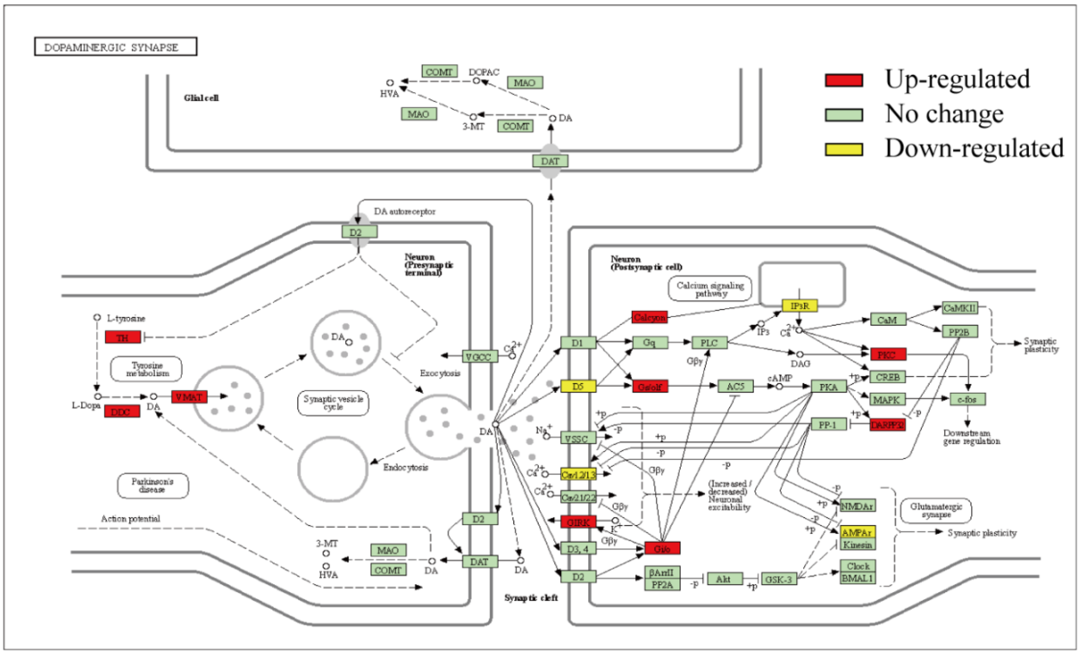

Fig. S2. Dopaminergic synaptic signal pathway analysis. (A) The differentially expressed genes in cells of 3D versus those of 2D in the dopaminergic synaptic signal pathway. (B) Dopaminergic synaptic signal pathway diagram by KEGG analysis.

Disease Models & Mechanisms • Supplementary information

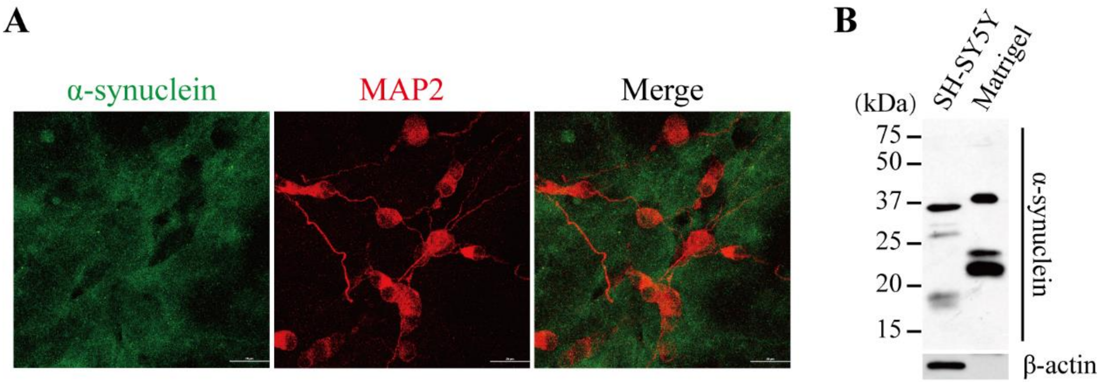

Fig. S3. Matrigel and α-synuclein. (A) Immunofluorescence staining of α-synuclein using the 3D constructs. Green, α-synuclein; red, MAP2. Bar size, 20 μm. (B) Western blot analysis of α-synuclein using SH-SY5Y cells and Matrigel, respectively.

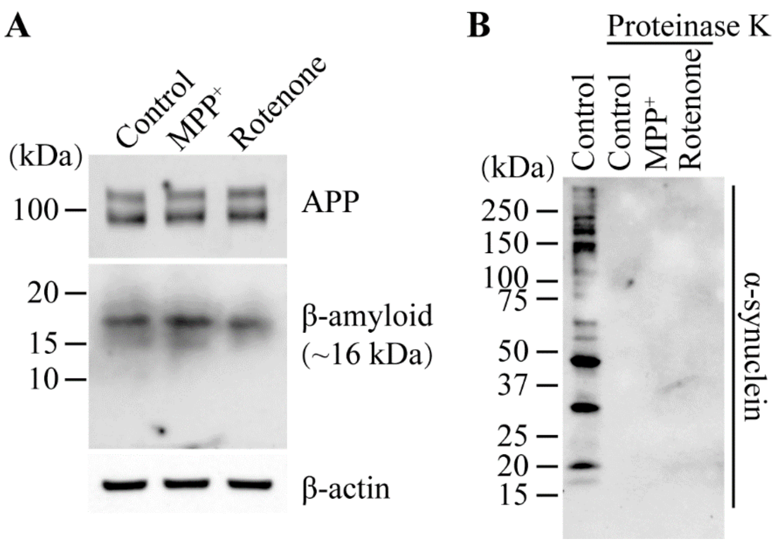

Fig. S4. Western blot analyses of β-amyloid (A) and proteinase K resistant α-synuclein (B) expression in the RA-treated 3D cultures. Cells were treated with 10 µM MPP^+ or 0.5 µM rotenone for 24 h. For (B), total cell lysates were treated with 5 µg/mL proteinase K at 37 °C for 30 min. APP, amyloid precursor protein; β-amyloid monomer is at about 4 kDa.

Table S1. Results of all statistical analyses undertaken

Figure #	Method	Results			
**Figure 1**	Factorial	Figure 1C			
	ANOVA	3D	RA	3D * RA+	
		p =< 0.0001	p =< 0.0001	p = 0.0175	
		2D	3D	RA-	RA+
		RA- vs RA+	RA- vs RA+	2D vs 3D	2D vs 3D
		p = 0.0002	p =< 0.0001	p =< 0.0001	p =< 0.0001
**Figure 2**	Student's	Figure 2B (2D vs 3D)			
	t-test	DDC	VMAT2		
		p = 0.0052	p = 0.0031		
**Figure 3**	Student's	Figure 3A			
	t-test	2D: α-syn			
		Ctrl vs MPP+		Ctrl vs Rotenone	
		monomer/LMW	HMW	monomer/LMW	HMW
		p = 0.0092	p = 0.1057	p = 0.0839	p = 0.2179
		3D: α-syn			
		Ctrl vs MPP+		Ctrl vs Rotenone	
		monomer/LMW	HMW	monomer/LMW	HMW
		p = 0.4711	p = 0.5539	p = 0.3098	p = 0.7274
		Figure 3B			
		2D: pS129-α-syn			
		Ctrl vs MPP+		Ctrl vs Rotenone	
		monomer/LMW	HMW	monomer/LMW	HMW
		p = 0.5166	p = 0.8379	p = 0.0916	p = 0.0095
		3D: pS129-α-syn			
		Ctrl vs MPP+		Ctrl vs Rotenone	
		monomer/LMW	HMW	monomer/LMW	HMW
		p = 0.0410	p = 0.0074	p = 0.0429	p = 0.0134
**Figure 4**	Student's	Figure 4A (2D: α-syn)		Figure 4B (3D: α-syn aggregates)	
	t-test	Ctrl vs MPP+	Ctrl vs Rotenone	Ctrl vs MPP+	Ctrl vs Rotenone
		p = 0.0042	p = 0.0636	p = 0.0039	p = 0.0010
		Figure 4C (3D: insoluble α-syn fraction)			
		Ctrl vs MPP+	Ctrl vs Rotenone		
		p =< 0.0001	p = 0.0032		
**Figure 5**	Student's	Figure 5A (3D: LB509+ cells)			
	t-test	Ctrl vs MPP+	Ctrl vs Rotenone		
		p = 0.0001	p = 0.0004		
**Figure 6**	Student's	Figure 6A (pS129-α-syn+ cells)		Figure 6B (Ubiquitin+ cells)	
	t-test	Ctrl vs MPP+	Ctrl vs Rotenone	Ctrl vs MPP+	Ctrl vs Rotenone
		p = 0.0430	p = 0.0350	p = 0.0342	p = 0.0466
		Figure 6C (β-amyloid+ cells)		Figure 6D (Thioflavin-S+ cells)	

		Ctrl vs MPP+<br>*p* = 0.0039	Ctrl vs Rotenone<br>*p* = 0.0017	Ctrl vs MPP+<br>*p* = 0.0001	Ctrl vs Rotenone<br>*p* = 0.0007
**Figure S1**	One-way ANOVA	Figure S1A			
		10% FBS	RA+ (FBS)		
		RA- vs RA+<br>*p* =< 0.0001	10% vs 5%<br>*p* = 0.7768	10% vs 3%<br>*p* = 0.9994	10% vs 2%<br>*p* = 0.6792
			10% vs 1%<br>*p* = 0.6708	5% vs 3%<br>*p* = 0.5994	5% vs 2%<br>*p* => 0.9999
			5% vs 1%<br>*p* => 0.9999	3% vs 2%<br>*p* = 0.4982	3% vs 1%<br>*p* = 0.4901
			2% vs 1%<br>*p* => 0.9999		
		Figure S1D			
		0 d vs 1 d<br>*p* = 0.0072	0 d vs 2 d<br>*p* =< 0.0001	0 d vs 3 d<br>*p* =< 0.0001	0 d vs 4 d<br>*p* =< 0.0001
		0 d vs 5 d<br>*p* = 0.0475	0 d vs 6 d<br>*p* = 0.6385	1 d vs 2 d<br>*p* = 0.0197	1 d vs 3 d<br>*p* = 0.0271
		1 d vs 4 d<br>*p* = 0.0378	1 d vs 5 d<br>*p* = 0.9358	1 d vs 6 d<br>*p* = 0.1430	2 d vs 3 d<br>*p* => 0.9999
		2 d vs 4 d<br>*p* => 0.9999	2 d vs 5 d<br>*p* = 0.0030	2 d vs 6 d<br>*p* = 0.0002	3 d vs 4 d<br>*p* => 0.9999
		3 d vs 5 d<br>*p* = 0.0041	3 d vs 6 d<br>*p* = 0.0002	4 d vs 5 d<br>*p* = 0.0057	4 d vs 6 d<br>*p* = 0.0003
		5 d vs 6 d<br>*p* = 0.5894			

α-syn, α-synuclein; Ctrl, control

Table S2. The differentially expressed genes in the 3D cultures compared to the 2D cultures by RNA-sequencing analyses (fold change > 2 and p < 0.05)

Click here to download Table S2

Disease Models & Mechanisms • Supplementary information
